# Association between wealth, insurance coverage, urban residence, median age and COVID-19 deaths across states in Nigeria

**DOI:** 10.1371/journal.pone.0291118

**Published:** 2023-09-08

**Authors:** Samuel A. Akinseinde, Samson Kosemani, Emmanuel Osuolale, Nina Cesare, Samantha Pellicane, Elaine O. Nsoesie

**Affiliations:** 1 The Amateur Polymath, Lagos, Nigeria; 2 Department of Immunology, Institute of Biomedical Sciences, University of São Paulo, São Paulo, Brazil; 3 Faculty of Engineering, Department of Marine Engineering, Rivers State University, Rivers State, Nigeria; 4 Biostatistics and Epidemiology Data Analytics Center, School of Public Health, Boston University, Boston, MA, United States of America; 5 Department of Epidemiology, School of Public Health, Boston University, Boston, MA, United States of America; 6 Department of Global Health, School of Public Health, Boston University, Boston, MA, United States of America; University of the Witwatersrand Johannesburg Faculty of Health Sciences, SOUTH AFRICA

## Abstract

This study measures associations between COVID-19 deaths and sociodemographic factors (wealth, insurance coverage, urban residence, age, state population) for states in Nigeria across two waves of the COVID-19 pandemic: February 27th 2020 to October 24th 2020 and October 25th 2020 to July 25th 2021. Data sources include 2018 Nigeria Demographic and Health Survey and Nigeria Centre for Disease Control (NCDC) COVID-19 daily reports. It uses negative binomial models to model deaths, and stratifies results by respondent gender. It finds that overall mortality rates were concentrated within three states: Lagos, Edo and Federal Capital Territory (FCT) Abuja. Urban residence and insurance coverage are positively associated with differences in deaths for the full sample. The former, however, is significant only during the early stages of the pandemic. Associative differences in gender-stratified models suggest that wealth was a stronger protective factor for men and insurance a stronger protective factor for women. Associative strength between sociodemographic measures and deaths varies by gender and pandemic wave, suggesting that the pandemic impacted men and women in unique ways, and that the effectiveness of interventions should be evaluated for specific waves or periods.

## Introduction

The coronavirus disease 2019 (COVID-19) pandemic has had an unprecedented impact on the health, economy, and quality of life on communities around the world [1]. As of late March 2022, over 474 million cases and 6 million deaths had been documented globally [2]. In Africa, about 5 million cases and over 115,000 deaths had been recorded [2]. Nigeria, the most populous country in Africa, had reported over 255,000 cases and over 3,100 deaths at this time [3].

Structural inequalities, vaccine apartheid and resource limitations are some factors affecting the COVID-19 response in sub-Saharan African countries [4]. While studies have evaluated public perceptions of pandemic risk and health protective behaviors in Nigeria [5], little is known about the factors that impact COVID-19 mortality in this country. Within rural areas, factors such as health center availability, transportation, and infrastructure challenges may render populations more vulnerable to disease [6]. While urban areas may benefit from accessible healthcare resources for detection and treatment, condensed residential areas may lead to higher transmission rates [7].

The aim of this study is to assess the association between specific place and sociodemographic factors (wealth, insurance coverage, urban residence, median age, gender) and COVID-19 deaths across states in Nigeria [8]. Our findings contribute to our understanding of COVID-19 dynamics in Nigeria and could support future pandemic planning.

## Material and methods

We used three datasets to complete this analysis. Two datasets are from the 2018 Nigeria Demographic and Health Survey (2018 NDHS) implemented by the National Population Commission (NPC) [9]. The third is from the Nigeria Centre for Disease Control (NCDC) COVID-19 daily reports [10]. The 2018 NDHS provides data on national demographic and public health indicators. Results are published separately for male and female residents, as biometric data are collected for female participants alone. Participants range in age from 15 to 59 years. For 2018 there were 41821 (75.9%) female and 13311(24.1%) male participants with an average of 1490 total participants per state.

The state-level sociodemographic factors include: wealth index, insurance coverage, median age, and type of residence. The wealth index was defined by NDHS based on household ownership of consumer goods (including, television, cars) and housing characteristics (such as, flooring materials and toilet facilities) [9]. We specifically focus on the percent of the population categorized by this index as ‘poorest’. Type of residence measures the percent of the population that lives within urban areas. Insurance coverage measures the percent of the population covered by health insurance. Given that each state is represented by a single, continuous age measure we elect to use median age. Median age is robust to outliers, which is appropriate given that a Shapiro-Wilk test indicates that current age within NDHS 2018 data is consistently, negatively skewed across states (p<0.001 for all). Furthermore, the position of median age indicates whether non-normality within a population is more likely attributable to births than aging.

The NCDC COVID-19 data includes daily reports of case counts, death counts and recovery counts for all 36 states and the Federal Capital Territory (FCT). The data used for this analysis were collected between February 27^th^, 2020, and July 25^th^, 2021. Since research has identified two ‘waves’ of COVID–one occurring between February 27^th^, 2020, and October 24^th^, 2020, and the second occurring between October 25^th^ 2020 and July 25th 2021 [11]–we split our sample at October 25^th^ 2020. These two periods are hereby referred to as period one and two. We calculate mortality rates as the number of deaths per 100,000 state residents. See Table 1 for a summary of all sociodemographic measures, populations, and COVID-19 deaths by state and political region.

**Table 1:** Sociodemographic characteristics and population by political region and state.

Political Region	State	Wealth index, poorest	Percent Insured	Median age	Percent urban residence	Percent watch TV 1+ time per week	Populationin tens of thousands	COVID- 19 Deaths (Full Period)	COVID- 19 Deaths (Period 1)
North Central	Benue	16.8	1.3	28	12.2	32.7	574.18	24	10
	FCT Abuja	5.3	12.6	29	59.5	43.5	356.41	168	79
	Kogi	2.6	1.5	28	31.5	32.4	447.35	2	2
	Kwara	19.0	5.2	29	68.2	26.1	319.29	55	25
	Nasarawa	3.6	1.7	28	25.2	28.1	252.34	39	13
	Niger	17.7	2.7	28	26.0	24.6	555.62	17	12
	Plateau	24.4	2.6	28	25.9	12.5	420.04	58	33
North East	Adamawa	17.8	1.1	28	26.6	17.5	424.84	32	19
	Bauchi	43.7	1.2	27	16.9	12.8	653.73	17	14
	Borno	28.6	1.6	27	37.7	14.5	586.02	38	36
	Gombe	37.9	0.7	27	25.0	18.5	325.70	44	25
	Taraba	30.7	1.3	27	18.1	15.3	306.68	24	6
	Yobe	66.7	1.1	26	25.1	14.9	329.41	9	8
North West	Jigawa	53.4	0.2	28	13.8	11.3	582.82	16	11
	Kaduna	5.6	4.3	29	47.8	28.8	825.24	65	42
	Kano	25.6	5.1	27	42.1	23.4	1307.69	110	54
	Katsina	19.1	0.5	26	24.5	13.5	783.13	34	24
	Kebbi	31.8	1.3	28	19.0	7.0	444.01	16	8
	Sokoto	43.3	8.8	28	28.9	12.5	499.81	28	17
	Zamfara	55.5	0.2	26	25.2	3.8	451.54	8	5
South East	Abia	0.0	8.0	32	23.1	56.6	372.73	22	8
	Anambra	0.2	1.7	30	81.2	54.5	552.78	19	19
	Ebonyi	16.9	0.7	31	83.9	17.8	288.04	32	30
	Enugu	1.6	1.5	30	74.5	29.1	441.11	29	21
	Imo	0.4	2.0	33	49.5	30.7	540.88	37	12
South South	Akwa Ibom	3.0	1.8	30	9.6	42.5	548.22	21	8
	Bayelsa	1.9	4.0	29	26.1	66.9	227.80	26	21
	Cross River	6.4	2.3	31	19.4	51.0	386.63	18	9
	Delta	1.0	1.6	30	49.4	47.0	566.34	72	49
	Edo	3.6	2.7	32	57.9	63.4	423.56	185	107
	Rivers	2.4	3.3	30	48.6	37.1	730.39	102	59
South West	Ekiti	6.7	2.7	30	73.5	46.5	327.08	11	6
	Lagos	0.0	6.0	32	94.8	81.8	1255.06	456	207
	Ogun	1.2	6.6	31	47.1	32.9	521.77	53	28
	Ondo	5.9	1.4	30	46.9	39.6	467.17	66	36
	Osun	8.6	1.4	30	75.6	79.6	470.56	52	20
	Oyo	4.8	3.4	32	75.8	51.9	784.09	127	40

### Statistical analyses

Models include factors likely to impact deaths counts, including population age, insurance coverage, wealth, urban residence, and total population. Prior to analysis, we tested each covariate for assumptions of normality. We also tested associations between covariates to ensure values did not far exceed a strong Pearson correlation. We noted a relatively strong association between median age and wealth for the full sample (*r =* 0.73) but we included in our model and assessed model fit with and without this covariate. We used median rather than mean age because this measure is robust to outliers, and because a Shapiro-Wilk test confirmed that the former is normally distributed across states.

We used negative binomial models to assess the relationship between state-level characteristics and COVID-19 deaths. For transparency, we allow our offset to remain at one and elect to control for population as an independent variable. Negative binomial models were selected due to the fact that the variance of deaths across the period considered (6325.9) far exceeded the mean number of deaths (57.6), and the dispersion parameter for the full model without this adjustment was 17.5. Likelihood ratio tests comparing negative binomial and Poisson models with all final covariates indicated the negative binomial is a better fit (*X*^*2*^ = 450, p<0.001). The selection of negative binomial models to account for overdispersion and correctly model COVID-19 death counts also corresponds with existing literature [12].

We used AIC as a primary determination for excluding or including covariates. We also assess the model fit after individually including and excluding variables that were significantly correlated with one another (i.e., *r* greater than or equal to 0.5). For example, while we found that median age was somewhat negatively correlated with our wealth index (*r =* -0.73), including this measure in our final model reduced the AIC value while preserving associative directionality for other measures. We elect to control for population as a state-level characteristic likely to impact deaths counts. While we considered including the percent of residents who watch TV at least once per week as a covariate given that media use may spread health-promoting messages [13], we elected to omit this from our analysis as it did not improve model fit.

Given that existing research documents COVID-19 incidence rates varying by gender [14], we present models for the overall sample, and for male and female samples separately. We present Incidence Rate Ratios in Table 2 and regression coefficients in Appendix Table 1 in S1 File. Significance is designated at p<0.05.

**Table 2:** Incidence rate ratios (95% CI) for negative binomial models evaluating associations between COVID-19 deaths and sociodemographic measures across states.

		Full sample		Female sample		Male sample	
		Incidence Rate Ratios (95% CI)	P-Value	Incidence Rate Ratios (95% CI)	P-Value	Incidence Rate Ratios (95% CI)	P-Value
Full time period: February 27^th^ 2020 to July 25^th^ 2021	Wealth index: Poorest, Percent	0.992 (0.974, 1.010)	0.328	0.991 (0.974, 1.009	0.282	0.981 (0.964, 0.999)	0.023
	Percent insured	1.092 (1.014, 1.188)	0.027	1.081 (1.007, 1.173)	0.036	1.060 (0.996, 1.136)	0.093
	Median age	1.050 (0.879, 1.261)	0.572	1.041 (0.884, 1.224)	0.618	0.937 (0.820, 1.065)	0.292
	Urban residences, percent	1.012 (0.999, 1.026)	0.025	1.014 (1.002, 1.027)	0.009	1.016 (1.002, 1.029)	0.008
	Population (in 10,000s)	1.001 (1.000, 1.002)	0.008	1.001 (1.000, 1.002)	0.009	1.001 (1.000, 1.002)	0.011
	Observations	37		37		37	
	Akaike Inf. Crit	351.230		351.94		351.67	
		Full sample		Female sample		Male sample	
		Incidence Rate Ratios (95% CI)	P-Value	Incidence Rate Ratios (95% CI)	P-Value	Incidence Rate Ratios (95% CI)	P-Value
Period one: February 27^th^ 2020 to October 24^th^ 2020	Wealth index: Poorest, Percent	0.995 (0.977, 1.013)	0.554	0.995 (0.978, 1.013)	0.557	0.985 (0.967, 1.003)	0.068
	Percent insured	1.069 (0.994, 1.161)	0.092	1.067 (0.993, 1.157)	0.080	1.033 (0.971, 1.103)	0.358
	Median age	1.010 (0.844, 1.213)	0.909	1.008 (0.858, 1.181)	0.919	0.920 (0.802, 1.050)	0.181
	Urban residences, percent	1.016 (1.003, 1.030)	0.003	1.017 (1.005, 1.030)	0.001	1.020 (1.006, 1.034)	0.001
	Population (in 10,000s)	1.001 (1.000, 1.002)	0.012	1.001 (1.000, 1.002)	0.011	1.001 (1.000, 1.002)	0.014
	Observations	37		37		37	
	Akaike Inf. Crit	308.700		308.545		309. 420	
		Full sample		Female sample		Male sample	
		Incidence Rate Ratios (95% CI)	P-Value	Incidence Rate Ratios (95% CI)	P-Value	Incidence Rate Ratios (95% CI)	P-Value
Period two: October 25^th^ 2020 to July 25^th^ 2021	Wealth index: Poorest, Percent	0.986 (0.959, 1.013)	0.234	0.984 (0.959, 1.011)	0.185	0.975 (0.951, 1.000)	0.031
	Percent insured	1.125 (1.013, 1.278)	0.034	1.102 (0.998, 1.239)	0.066	1.104 (1.006, 1.229)	0.040
	Median age	1.098 (0.853, 1.430)	0.441	1.089 (0.855, 1.388)	0.461	0.956 (0.795, 1.140)	0.604
	Urban residences, percent	1.006 (0.987, 1.025)	0.414	1.009 (0.991, 1.027)	0.232	1.009 (0.990, 1.028)	0.283
	Population (in 10,000s)	1.001 (1.000, 1.002)	0.047	1.001 (1.000, 1.002)	0.051	1.001 (1.000, 1.002)	0.059
	Observations	37		37		37	
	Akaike Inf. Crit	304.097		305.214		303.675	

## Results

**Fig 1:**
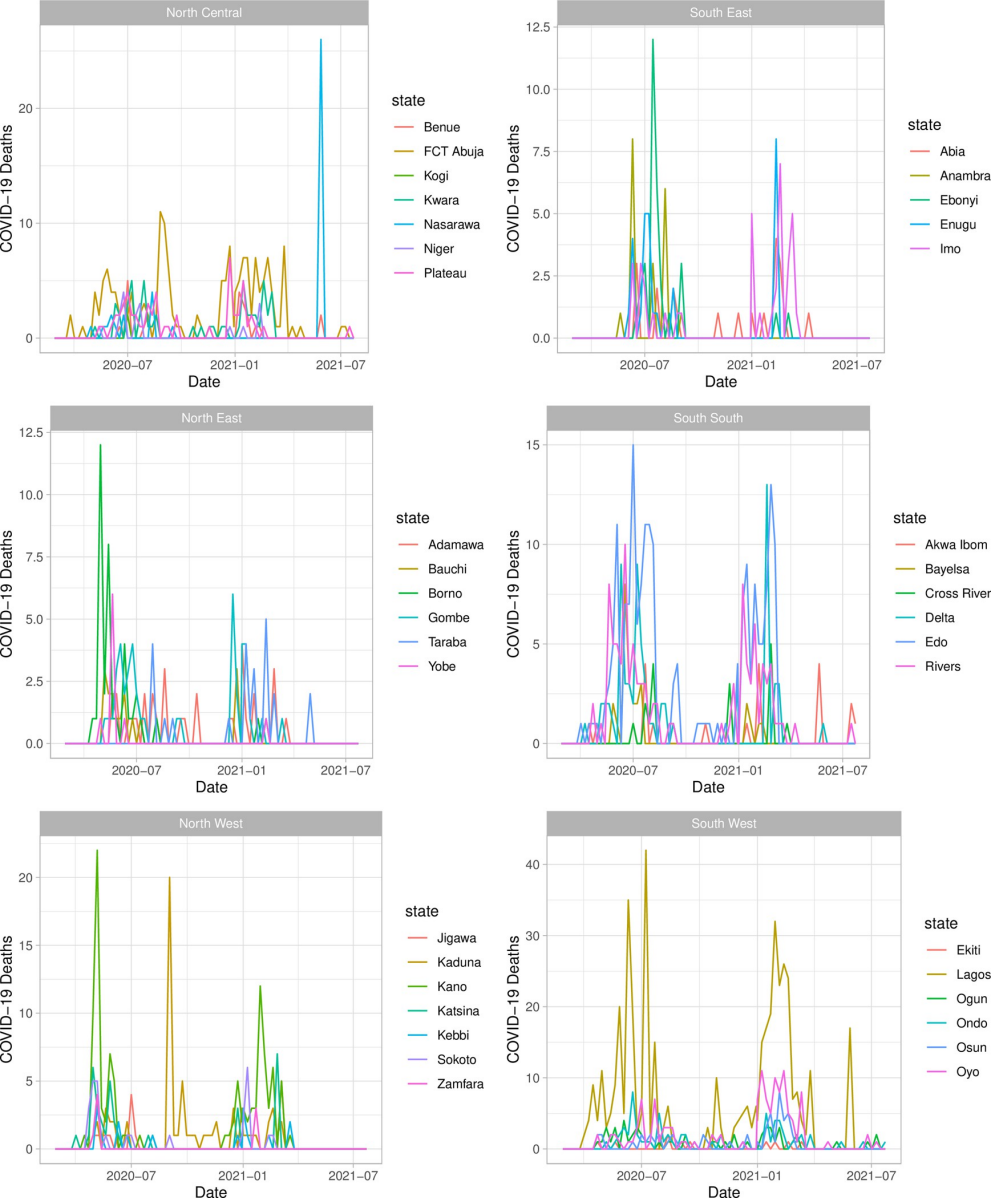
Total COVID-19 deaths by week for each state in Nigeria by week, organized by political regions.

Between February 27th, 2020, and July 25th, 2021, Nigeria reported 2,132 COVID-19 deaths. Period one (February 27^th^, 2020, to October 24^th^, 2020) saw 1123 deaths compared to 1009 deaths during period two (October 25^th^, 2020, to July 25^th^, 2021). Fig 1 displays the total deaths occurring within each state by week for the full time period.

**Fig 2:**
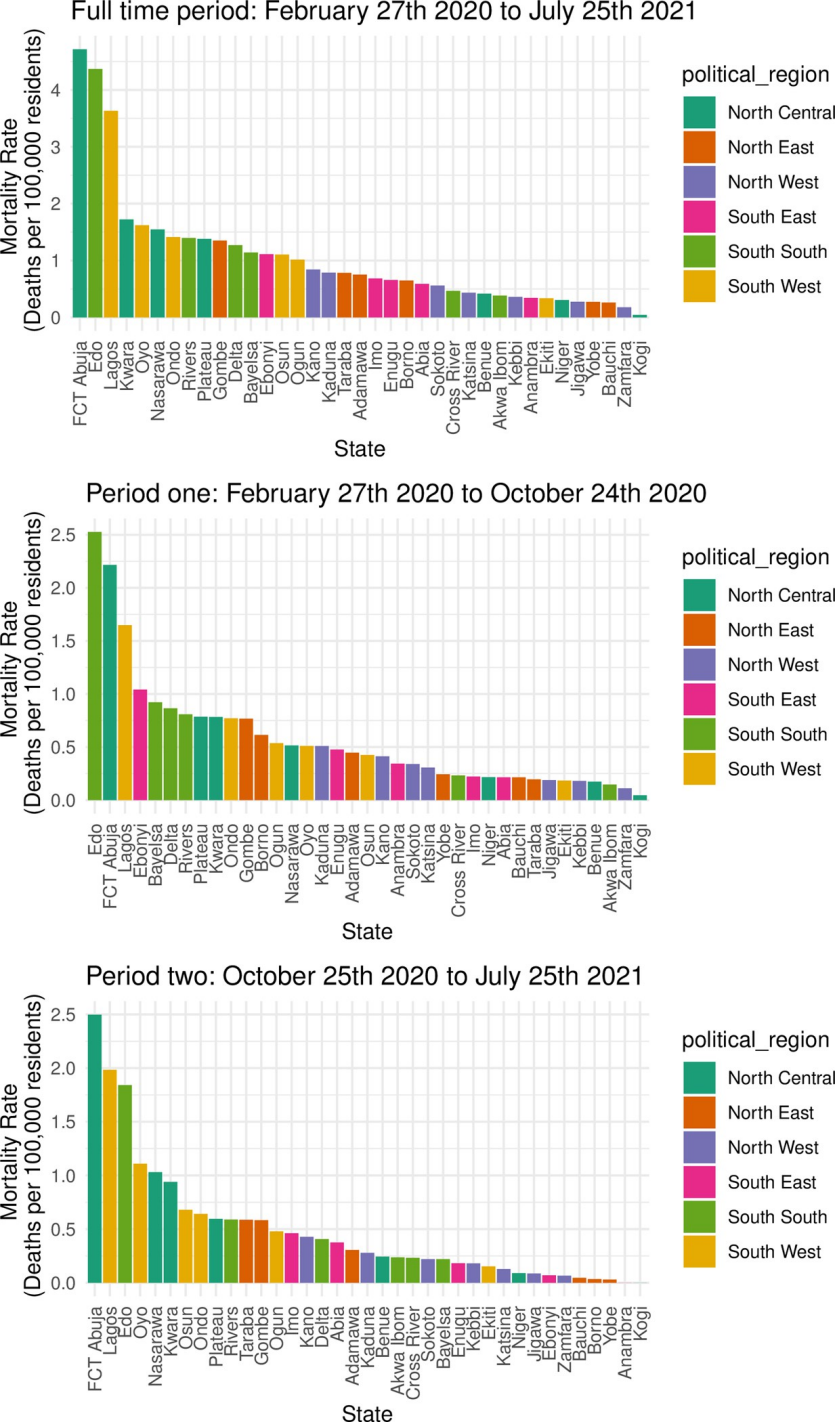
Mortality rates (deaths per 100,000 residents) by state and region for each time period analyzed.

Trends in mortality varied by state for each period analyzed (Fig 2). Furthermore, there was no consistent pattern between mortality and political region. Lagos state reported the largest number of deaths for the full period (456), period one (207) and period two (249). However, mortality rates were concentrated among three states: FCT Abuja (4.7 per 100,000 residents for the full period, 2.2 per 100,000 residents for period one, and 2.5 per 100,000 residents for period two), Edo (4.5 per 100,000 residents for the full period, 2.5 per 100,000 residents for period one, and 1.8 per 100,000 residents for period two), and Lagos (3.6 per 100,000 residents for the full period, 1.6 residents for period one, and 2.0 residents for period two).

Table 2 displays incidence rate ratios for negative binomial models evaluating associations between COVID-19 deaths and sociodemographic measures across states. Coefficients are displayed in Appendix Table 1 in S1 File. The percent of the state categorized as urban is also significantly associated with COVID-19 deaths overall and during period one. During period one, a one percent increase in urban residents results in 1.0 times as many deaths. These patterns are consistent across gender-stratified samples.

While insurance coverage across states is limited (average is 2.8% with a maximum of 12.6% in the FCT), percent insured is significantly, positively associated with death counts across states. For the full sample, this association is particularly strong during period two, where a one percent increase in insurance coverage leads to 1.1 times as many deaths (95% CI 1.0 to 1.2).

Insurance coverage is significant for the female sample across the full time period but is significant for the male sample only during period two. The effect of wealth also varies by gender. Wealth is a significant predictor of additional deaths within the male sample, but not the female or overall sample.

While an increase in median age is associated with an increase in mortality, this effect is not significant during either period. Similarly, states with lower wealth index values reported fewer COVID-19 deaths but this association is not significant during either time period.

## Discussion

While the highly populated state of Lagos saw the largest number of COVID-19 deaths, Edo, FCT Abuja and Lagos experienced the highest mortality rate during the pandemic. Controlling for population, we observe that urban residence and insurance coverage are positively and significantly associated with COVID-19 deaths across states in Nigeria. Taken together, these findings suggest that urban density might be a major driver of COVID-19 deaths reported in Nigeria. This may help support pandemic planning during future COVID-19 waves or other infectious disease epidemics, and may be indicative of patterns in other countries with similar sociodemographic profiles [15].

Furthermore, we note that the significance of sociodemographic factors and geographic concentration of COVID-19 deaths varies depending on the pandemic wave considered. Research examining COVID-19 incidence over time has found similar time-variant effects for sociodemographic predictors [16]. This suggests that pandemic-related programs may be re-evaluated based on significant drops or increases in incidence rates.

Associative differences in gender-stratified models suggest that social conditions impacted male and female vulnerability differently during the pandemic. Wealth was a stronger protective factor for men than women. Men who lived in wealthier areas saw more positive COVID outcomes. Insurance coverage was a stronger protective factor for women during period one. It is possible that women in areas with higher insurance coverage experienced other protective factors ‐ such as stable housing and employment ‐ during the first COVID wave.

Identifying sociodemographic risk factors for COVID-19 deaths is particularly important for nations like Nigeria, which did not see the same severe, initial outbreak trajectories observed in many other countries despite the fact that restrictions at the start of the pandemic were fairly lax [17]. The unique trajectory of COVID-19 in this country invites new inquiry into the regional, social and political factors associated with the spread and impact of infectious disease.

This study is limited by the availability of state-specific, up-to-date sociodemographic information and small-area COVID-19 data. The development of small-area infectious disease monitoring and demographically comprehensive sociodemographic data collection would greatly improve the ability to identify which areas of Nigeria are vulnerable to outbreaks. Additionally, due to the challenge of identifying reliable sources of state-level data, we cannot control for health-protective policy changes. Additionally, using state-level data renders us subject to ecological bias; patterns observed at the state level may not be true of individuals [18]. Finally, we are limited by possible underreporting of cases and deaths. Seroprevalence surveys and postmortem tests suggest reported COVID-19 cases are many folds lower than actual cases across many contexts [19–21], and it is possible that our data are susceptible to similar errors.

## Supporting information

S1 File (DOCX)
