# Supplementary material for: Association between wealth, insurance coverage, urban residence, median age and COVID-19 deaths across states in Nigeria
Source: PLoS One. 2023 Sep 8;18(9):e0291118. doi: 10.1371/journal.pone.0291118 (PMC10490894; doi:10.1371/journal.pone.0291118)
Supplement: S1 File — (DOCX) [file pone.0291118.s001.docx]

APPENDIX

Table 1: Negative Binomial Coefficients Evaluating Associations between COVID-19 Deaths and Sociodemographic Measures across States

|  |  | Full sample | | Female sample | | Male sample | |
| --- | --- | --- | --- | --- | --- | --- | --- |
|  |  | Coef. (SE) | P-Value | Coef. (SE) | P-Value | Coef. (SE) | P-Value |
| Full time period: February 27^th^ 2020 to July 25^th^ 2021 | Wealth index: Poorest, Percent | -0.008 (0.009) | 0.328 | -0.009 (0.008) | 0.282 | -0.019 (0.008) | 0.023 |
|  | Percent insured | 0.088 (0.040) | 0.027 | 0.078 (0.037) | 0.036 | 0.058 (0.035) | 0.093 |
|  | Median age | 0.049 (0.087) | 0.572 | 0.041 (0.081) | 0.618 | -0.065 (0.062) | 0.292 |
|  | Urban residences, percent | 0.012 (0.005) | 0.025 | 0.014 (0.005) | 0.009 | 0.016 (0.006) | 0.008 |
|  | Population (in 10,000s) | 0.001 (0.000) | 0.008 | 0.001 (0.000) | 0.009 | 0.001 (0.000) | 0.011 |
|  | Intercept | 1.108 (2.553) | 0.664 | 1.372 (2.352) | 0.560 | 4.704 (1.988) | 0.018 |
|  | Observations | 37 | | 37 | | 37 | |
|  | Log likelihood | -337.229 | | -337.944 | | -337.673 | |
|  | Akaike Inf. Crit | 351.230 | | 351.94 | | 351.67 | |
|  |  | Coef. (SE) | P-Value | Coef. (SE) | P-Value | Coef. (SE) | P-Value |
| Period one: February 27^th^ 2020 to October 24^th^ 2020 | Wealth index: Poorest, Percent | -0.005 (0.009) | 0.554 | -0.005 (0.008) | 0.557 | -0.015 (0.008) | 0.068 |
|  | Percent insured | 0.067 (0.040) | 0.092 | 0.065 (0.037) | 0.080 | 0.032 (0.035) | 0.358 |
|  | Median age | 0.0109 (0.087) | 0.909 | 0.008 (0.081) | 0.919 | -0.084 (0.063) | 0.181 |
|  | Urban residences, percent | 0.016 (0.005) | 0.003 | 0.017 (0.005) | 0.001 | 0.020 (0.006) | 0.001 |
|  | Population (in 10,000s) | 0.001 (0.000) | 0.012 | 0.001 (0.000) | 0.011 | 0.001 (0.000) | 0.014 |
|  | Intercept | -1.531 (2.574) | 0.552 | 1.571 (2.343) | 0.502 | 4.597 (2.013) | 0.022 |
|  | Observations | 37 | | 37 | | 37 | |
|  | Log likelihood | -294.701 | | -294.545 | | -295.420 | |
|  | Akaike Inf. Crit | 308.700 | | 308.545 | | 309. 420 | |
|  |  | Coef. (SE) | P-Value | Coef. (SE) | P-Value | Coef. (SE) | P-Value |
| Period two: October 25^th^ 2020 to July 25^th^ 2021 | Wealth index: Poorest, Percent | -0.014 (0.012) | 0.234 | -0.016 (0.012) | 0.185 | -0.025 (0.012) | 0.031 |
|  | Percent insured | 0.118 (0.056) | 0.034 | 0.097 (0.053) | 0.066 | 0.099 (0.048) | 0.040 |
|  | Median age | 0.094 (0.122) | 0.441 | 0.085 (0.115) | 0.461 | -0.045 (0.086) | 0.604 |
|  | Urban residences, percent | 0.006 (0.008) | 0.414 | 0.009 (0.007) | 0.232 | 0.009 (0.008) | 0.283 |
|  | Population (in 10,000s) | 0.001 (0.001) | 0.047 | 0.001 (0.001) | 0.051 | 0.001 (0.001) | 0.059 |
|  | Intercept | -0.813 (3.591) | 0.821 | -0.493 (3.337) | 0.883 | 3.442 (2.776) | 0.215 |
|  | Observations | 37 | | 37 | | 37 | |
|  | Log likelihood | -290.097 | | -291.214 | | -289.675 | |
|  | Akaike Inf. Crit | 304.097 | | 305.214 | | 303.675 | |
